# The Use of Intrinsic Markers for Studying the Migratory Movements of Bats

**DOI:** 10.3390/ani11123477

**Published:** 2021-12-06

**Authors:** Caralie T. Brewer, William A. Rauch-Davis, Erin E. Fraser

**Affiliations:** School of Science and the Environment, Grenfell Campus, Memorial University of Newfoundland, 20 University Drive, Corner Brook, NL A2H 5G4, Canada; caralieb@grenfell.mun.ca (C.T.B.); wrauchdavis@grenfell.mun.ca (W.A.R.-D.)

**Keywords:** intrinsic markers, Chiroptera, wind energy, stable isotopes, radiogenic isotopes, trace elements, contaminants, paired techniques, metabolically active/inert, tissue turnover

## Abstract

**Simple Summary:**

Migratory bats species are among the most heavily impacted by the erection of wind energy facilities, with many individuals killed at wind turbines each year. Bat carcasses may be collected and used for a variety of biological studies. In this paper, we review the use of intrinsic markers—chemical signatures in bat tissues that can provide information about that animal’s life history—to study bat movements across the landscape. In doing so, we aim to provide our audience with a better understanding of the currently available literature and, more importantly, the areas of this field that need expansion. We emphasize the applications of intrinsic markers that have not been used extensively to study migratory bat species (i.e., trace elements, contaminants, strontium isotopes), and provide a workflow for researchers interested in conducting studies of this type.

**Abstract:**

Mortality of migratory bat species at wind energy facilities is a well-documented phenomenon, and mitigation and management are partially constrained by the current limited knowledge of bat migratory movements. Analyses of biochemical signatures in bat tissues (“intrinsic markers”) can provide information about the migratory origins of individual bats. Many tissue samples for intrinsic marker analysis may be collected from living and dead bats, including carcasses collected at wind energy facilities. In this paper, we review the full suite of available intrinsic marker analysis techniques that may be used to study bat migration, with the goal of summarizing the current literature and highlighting knowledge gaps and opportunities. We discuss applications of the stable isotopes of hydrogen, oxygen, nitrogen, carbon, sulfur; radiogenic strontium isotopes; trace elements and contaminants; and the combination of these markers with each other and with other extrinsic markers. We further discuss the tissue types that may be analyzed for each and provide a synthesis of the generalized workflow required to link bats to origins using intrinsic markers. While stable hydrogen isotope techniques have clearly been the leading approach to infer migratory bat movement patterns across the landscape, here we emphasize a variety of lesser used intrinsic markers (i.e., strontium, trace elements, contaminants) that may address new study areas or answer novel research questions.

## 1. Introduction

Globally, many bat species move substantially across the landscape [1], although the dominant patterns in these movements are understudied. Many species engage in swarming and hibernation behaviors at sites distinct from their summer roosts [1,2,3,4,5,6,7], and the associated movements among these sites are frequently at a regional scale, with some long-distance movements consistently reported (if apparently rare) [3,8,9,10,11]. Other species have more frequently been reported migrating long distances (>1000 km); these movements may be to and from hibernation sites [12,13,14,15] or following ephemeral food resources [16,17]. At least some members of several North American species migrate substantially across latitudes, e.g., [18,19,20], likely to the more temperate overwintering locations and perhaps to forgo hibernation, but see [21]. There is substantial evidence for partial or differential migratory systems within bat species [22], with variation among populations, sexes, and individuals of various species. 

Protection of significant habitat is a key component in wildlife conservation. The paucity of knowledge about bat movements makes identification of significant habitat for these increasingly imperiled species challenging. The difficulties in tracking migratory movements of individual bats are well-documented. The small size and nocturnal nature of insectivorous bat species present challenges associated with capturing and re-capturing individuals, making the effective use of most extrinsic marking techniques problematic e.g., [23,24]. More recent technological innovations such as small-size satellite tags [21] and radio telemetry arrays [8,25,26,27] have increased the potential for tracking bat migration but have so far been used rarely. Further, such techniques still require a capture and release event, which is resource intensive.

A suite of techniques for tracking animal movements based on intrinsic markers (“biomarkers”) also exist and have been applied to investigations of bat movement systems. The guiding principle for most of these techniques is that bats incorporate various naturally occurring chemical signatures into their tissues, and these signatures are representative of the landscape where the tissue was formed. Examples of intrinsic markers include the stable isotope composition of “light” isotopes of elements such as hydrogen, oxygen, carbon, nitrogen, and sulfur; “heavy” isotopes of elements such as strontium and lead; and the relative compositions of trace elements and contaminants. Given a comprehensive understanding of chemical variation across the landscape, such signatures can be used to make origin assignment estimates of migratory individuals. 

One benefit of intrinsic marking techniques is that they can be used on tissues from pre-deceased animals, such as those in museum collections [20,28,29,30] or those killed at wind energy facilities [19,31,32]. The bats most frequently killed by wind turbines are those species typically considered long distance or latitudinal migrants [32,33,34]. Mortality rates of *Lasiurus cinereus* over the past two decades are likely leading to unsustainable population declines in this species [35,36]. The substantial number of bats collected under wind turbines each year provide a valuable source of tissue samples that may contribute to, among other uses, intrinsic marker analyses. These can aid in further elucidation of the origins and associated migratory patterns of highly mobile bat species.

Intrinsic markers provide an indirect source of data about animal origin, and thus require researchers to make a series of assumptions and interpolations e.g., [37]. For example, researchers must consider the natural variation of chemical markers in the environment, the mechanisms through which the markers are incorporated into animal tissues, the resulting concentration of the marker of interest, and the timing of tissue formation and turnover. Further, there is often an offset between the value (“signature”) of the intrinsic marker in the tissue and that in the local environment (requiring a transfer/rescaling function). Limitations in these steps and analytical processing have restricted the use of the full suite of intrinsic markers for investigations of bat biology. There is a substantial body of literature detailing the use of stable hydrogen isotope analyses of fur to investigate bat migration, although there is still much to be achieved in this area. Studies using other types of intrinsic markers are less frequent in the field of bat migration science, but see [38,39,40,41], as are studies that use samples from non-fur tissues, but see [41,42,43]. Further development in this field will improve our ability to learn about the biology of individual migratory bats. Combinations of different intrinsic marker analyses on tissues samples from the same bat can narrow origin estimates [40] and analyses of various tissues can provide information about different times in an individual bat’s life, e.g., [41,42,43]. 

The over-arching goal of this paper is to review the full suite of available intrinsic marker analyses, and the tissue types that may be analyzed for each, within the framework of investigating bat movement systems. Our objectives are threefold. Firstly, (i) we will describe the types of intrinsic markers that may be used to learn about migrant origin and summarize the body of literature that has used these markers to study bat migration, highlighting areas for future research; (ii) we will further list the tissues that may be used for various intrinsic marker analyses and describe bat-specific considerations for each; and finally, (iii) we will provide a synthesis of the generalized workflow required to use intrinsic markers for linking bats to origins with an emphasis on identifying research (and knowledge gaps) that explicitly address that workflow. Because there is already a significant body of literature dedicated to reviewing the use of stable carbon, nitrogen, and hydrogen isotopes for studying bat migration [44], we provide a more cursory treatment of these and a more detailed focus on the lesser used markers.

## 2. Intrinsic Markers in Studies of Bat Migration

### 2.1. Using the Stable Isotopes of Hydrogen and Oxygen to Study Bat Migration

Predictable, continental-scale variation in the stable hydrogen and oxygen isotope compositions of precipitation make these markers particularly well suited for investigations of long-distance migration systems. Stable hydrogen isotope techniques are widely used to study the migratory systems of extant animals, usually through analyses of keratinous (feathers and fur) or chitinous (insect) tissues, and several reviews deal with this topic, e.g., [37,45,46]. Stable oxygen isotope techniques can also be informative in movement studies but are most frequently used for this purpose in modern or paleoecological studies using analyses of calciferous tissues, e.g., [47,48]. The heavy isotopes of both hydrogen and oxygen preferentially condense through Rayleigh distillation [49] when precipitation forms from meteoric water vapor. Therefore, the stable hydrogen and oxygen isotope compositions of precipitation vary accordingly with continental climatic patterns, including with latitude in many parts of the world, with season and temperature, across elevation, with distance from the coast, and with relative humidity [50]. 

Sources of hydrogen in animal tissues include diet and environmental water. The stable hydrogen isotope composition of tissues (δ^2^H_tissue_) is governed by complex physiological processes including both catabolic processes and evaporative water loss [51,52]. There is some evidence for a trophic effect on the δ^2^H values of animal proteins, e.g., [53], but the role of δ^2^H values as a trophic marker is still under investigation [51]. There is substantial evidence for variation in the δ^2^H values of organisms with aquatic- and terrestrial-based diets, e.g., [54]. Different bat species may route water from different sources (i.e., insectivorous bats may source more hydrogen from environmental water compared to frugivorous bats which may source water from their diet) [55]. In addition to food and water sources, δ^18^O_tissue_ has the third influence of inhaled O_2_. The proportion of δ^18^O_tissue_ derived from inhaled O_2_ depends on the volume of drinking water consumed [56], with implications for the relationship between δ^18^O_precip_ and δ^18^O_tissue_. Additionally, fractionation of the stable isotopes of oxygen among trophic levels is complicated by the many sources (i.e., drinking water, diet, oxygen in breath), and terminuses of oxygen (i.e., exhaled breath, urine, feces) in a single individual [57]. 

Stable hydrogen isotope composition is the most frequently used intrinsic marker to study bat migration and most studies have focused on North American and European migratory systems, but see [30]. The continental scale variation of δ^2^H_precip_ values is most suited for research questions investigating largescale movements across latitudes. Some research on bird migratory systems have successfully used stable hydrogen isotope techniques to detect smaller scale movements of organisms across elevations, e.g., [58,59], but attempts to do this with bats have so far had limited success [40,60,61]. Researchers have used stable hydrogen isotope techniques to estimate the origins of individual bats captured or collected at important features such as wind energy facilities, e.g., [31,34], or hibernacula, e.g., [62,63]. Others seek to identify continental scale patterns in migratory movements by sampling bats across locations, often using museum specimens, e.g., [28,29,30]. Research in both the U.S. and Germany has shown that bat fatalities at wind energy facilities include both local and migratory bats in varying proportions [19,31,32]. Research at hibernacula and swarming sites has identified sites with greater and lesser catchment areas [62,64] and has revealed changes over time in bat migratory habits [63]. There is clear evidence that many bat species have partial and differential migratory patterns, with variation among sexes [19,20,29,63], age groups, e.g., [65,66], and among bats with varying anatomy [63]. 

The transfer function linking the stable hydrogen isotope composition of bat fur to that of local precipitation has also been developed for many individual species (Appendix A) [67], as well as generically using data from sedentary species [30,40]. The use of species-specific transfer functions is ideal, as interspecific variation in these functions may impact origin assignment [67]. While there are strong transfer functions for many species, there is still substantial variation in the δ^2^H_fur_ values of bats within and among species at common locations [68,69]. Mean δ^2^H_fur_ values may vary significantly among proximate roosts [55,69] and reproductive females may have fur that is depleted of ^2^H compared to juveniles [66,69]. Variation among species can be even more significant. Voigt et al. [55] reported 65‰ variation among neotropical bats of 36 species and much of this variation may be caused by a trophic effect of discrimination [55,60,70], as well as substantial differences in the δ^2^H_fur_ values of bats consuming aquatic and terrestrial prey [54]. Understanding the sources of variation in the δ^2^H_fur_ values of resident bats can improve both the accuracy and precision of origin estimates, e.g., [66,67]. 

Stable hydrogen isotope analyses of organic tissues are limited by the presence of a proportion of hydrogen that is exchangeable with atmospheric water vapor [71,72]. All samples must be analyzed alongside matrix-matched standards with known non-exchangeable δ^2^H values. Samples and standards must be treated identically throughout preparation and analysis, including an equilibration process. Currently, internationally recognized keratin samples exist [73], and standards for other tissue types must be developed in house. As a result, most studies use keratinous tissues; usually fur, but occasionally claws [70]. Recent work on monarch butterflies has used the stable hydrogen isotope composition of lipids (which have no exchangeable hydrogen) to investigate animal origin [74], and there is further much potential for the compound-specific analyses of the stable hydrogen isotope composition of fatty acids [75]. This is surely an area for future development in bat research. 

Stable oxygen isotope techniques have not been widely used to investigate bat migration. In temperate climates, the δ^18^O signature varies with precipitation type (i.e., snow vs. rain) and thus forms predicable seasonal variation in tissues such as teeth and bones [56]. Stable oxygen isotope techniques are used most extensively in multi-isotope studies investigating movements and the life histories of extant and extinct ungulates, e.g., [47,48,76,77], as well as to explore land use strategies in other modern mammals, e.g., [78]. Although some researchers have sought to use δ^18^O to study migratory bird movements [79,80,81,82], there is a relatively weak correlation between δ^18^O_precip_ and δ^18^O_feathers_ compared to δ^2^H [82]. Additionally, there is no international keratin standard for stable oxygen isotopic analysis [82]. For more information on δ^18^O analytical methods, see Appendix B, Table A1.

### 2.2. Using the Stable Isotopes of Carbon and Nitrogen to Study Bat Migration

Stable carbon and nitrogen isotope techniques are used widely in studies of bat biology, most often inferring diet and habitat use, e.g., [83,84,85,86,87]. While many of these studies have examined stable isotope signatures in bat tissues, there is also a significant body of literature that describes stable isotope analyses of contemporary and subfossil bat guano collected beneath roosting colonies, e.g., [88], usually to investigate paleoenvironment [89]. There is further a growing amount of literature investigating the stable carbon isotope signature of bat breath, e.g., [90]. Applications using stable carbon and nitrogen isotope compositions for studying migration systems are limited by the lack of predictable largescale variation in the markers of interest across the landscape. Ecosystem variation in δ^13^C is largely driven by variation in trophic level [91], as well as in the δ^13^C_tissue_ values of plants with different photosynthetic pathways [92]. δ^15^N_tissue_ values vary predictably with trophic level [91] and other physiological characteristics of individual organisms [93]. As a result, these two markers are typically most effectively used to infer movement among locations where there is known variation in the prevalence of C3, C4 and Crassulacean Acid Metabolism (CAM) photosynthesizing plants (δ^13^C), e.g., [94,95] or variation in other environmental factors (e.g., drought) [96]. Both markers are frequently used in combination with other markers in studies of migration [95,97,98]. 

Fleming et al. [17] conducted the first study investigating bat migratory movements using stable isotope techniques and successfully identified migratory movements of nectarivorous *Leptonycteris curasoae* based on dietary shifts between C_3_ and CAM plants as the bats moved across landscape. Segers and Broders [64] used stable carbon and nitrogen isotopes to identify highly variable summer origins of bats captured at swarming sites in Nova Scotia, Canada. Other applications of stable carbon and nitrogen isotope techniques to study bat movement have used them in combination with other stable light isotopes e.g., [40,41,65]. In a multi-isotope study, Voigt et al. [61] found that the stable isotopes of nitrogen and carbon were particularly useful in tracking seasonal elevational movements of *Miniopterus natalensis* at Mount Kilimanjaro [61]. 

### 2.3. Using the Stable Isotopes of Sulfur to Study Bat Migration

Stable sulfur isotopes are among the lesser used stable light isotopes for studying animal ecology and have been used infrequently to study bat movements. Four stable isotopes of sulfur exist, but the most common ratio studied is ^34^S/^32^S (or δ^34^S), reported relative to the international standard, Vienna Canyon Diablo Troilite (VCDT). Sources of sulfur in the environment include (1) the oceans, i.e., oceanic sediments and seawater, (2) soils and lithology (depending on rock type and age), (3) the atmosphere, in the form of dust, pollution, and sea spray, (4) freshwater aquatic environments, and (5) biological materials, i.e., decaying organic matter and fossil fuels [99]. Additionally, agricultural landscapes may influence the local δ^34^S signature, as sulfur is a common soil amendment in both inorganic and organic fertilizers [78]. δ^34^S is incorporated into organic tissues via amino acids, most commonly in cysteine and methionine, which both have sulfur in their side chains [99]. The δ^34^S composition of many tissues has been studied, e.g., [100], and the method of δ^34^S incorporation is often related to the tissue’s amino acid content. Sulfur incorporation into fur and feathers, specifically, is well understood because keratin is a structural protein and therefore contains relatively large amounts of sulfur (up to 5%) [99]. Additionally, there is minimal fractionation of δ^34^S between trophic levels [99]. Due to the abundance of sulfur in fur keratin and the resulting small sample needed for analysis (Appendix B, Table A2), δ^34^S can be easily incorporated into intrinsic marking studies of bat migration. 

Stable sulfur isotopes are less common than other intrinsic markers in studies of migration because the variation of δ^34^S across the terrestrial landscape is still largely undescribed, but see [101], and the analytical methodology is not standardized (Appendix B, Table A2), see [57]. Stable sulfur isotopes are most commonly used in combination with ^87^Sr/^86^Sr in archaeological studies, e.g., [102,103,104] and δ^2^H, δ^15^N, and δ^13^C in studies of modern migratory vertebrates, e.g., [78,105,106,107]. We know of only two studies that have incorporated δ^34^S into studies of bat movement ecology and neither specifically used δ^34^S to investigate migration. Cryan et al. [68] used δ^2^H, δ^15^N, δ^13^C, and δ^34^S to investigate habitat use and prey selection of two roosting colonies of *Eptesicus fuscus*. Later, Dechmann et al. [108] used radio telemetry in combination with δ^34^S, δ^13^C, and δ^15^N analysis of fur and feces to investigate differences in diet, foraging behavior, and body condition between sexes of *Nyctalus noctula*. 

Studies which have solely used δ^34^S to map domestic livestock movements (i.e., sheep, cattle) across the landscape have been successful e.g., [109,110], and there is great potential to expand these techniques to investigate bat migration. Due to the distinct and uniform marine δ^34^S signature (+20.3‰) [99], many studies have used stable sulfur isotopes to differentiate between marine and terrestrial origin, e.g., [105,109,111], and this has clear application to bat migration systems. For example, Cryan et al. [19] provided evidence that some *L. cinereus* migrate longitudinally between inland and coastal areas. Stable sulfur isotope analyses of fur samples from this species could further investigate this assertion. Similarly, tracking migrations along coastlines via proximity to sea is important for some European bat species that may be impacted by offshore wind energy facility development e.g., [39]. Studies of other taxa also utilize other sources of variation in δ^34^S across the terrestrial landscape, including lithology, e.g., [106,110], and agricultural fertilizers, e.g., [78]. A significant knowledge gap is the need to describe δ^34^S variation across landscapes in tissues of sedentary bat species or using known-origin individuals. 

### 2.4. Using Strontium Isotopes to Study Bat Migration

Strontium isotopes (^87^Sr/^86^Sr) are radiogenic, meaning they are formed by the decay of a secondary element. The relatively heavy isotope ^87^Sr is formed when an isotope of rubidium (^87^Rb) radioactively decays see [112]. Both strontium and rubidium can substitute for calcium and potassium, respectively, in minerals [113]. Therefore, ^87^Sr/^86^Sr signatures in the landscape are largely related to (1) the ^87^Sr/^86^Sr concentration in the underlying geology, considering the age of rock and ^87^Rb, ^86^Sr, and ^87^Sr concentrations at the time of formation; (2) the ^87^Sr/^86^Sr concentration in the soil; (3) ^87^Sr/^86^Sr in the atmosphere in the form of dust, pollution, or sea spray; and (4) the ^87^Sr/^86^Sr concentration of surface waters [112,113,114]. Biological incorporation of these strontium isotopes occurs through the diet and water consumption of the organism of interest [115,116].

Researchers in the fields of palaeoecology and archaeology have extensively used strontium isotopic patterns in the landscape to delineate prehistoric movements of various species, e.g., [48,117] including humans (although that is beyond the scope of this review, see [112,115,118]). These studies commonly analyze calciferous tissues (e.g., bones and teeth), which have relatively high concentrations of Sr, with Sr^2+^ substituting for Ca^2+^ in those tissues. Strontium isotope techniques have rarely been used to study migration of modern aerial vertebrates but see [39,119,120], likely because of multiple challenges associated with the technique. The method for strontium incorporation into keratin is not well understood, but see [121], and the relative concentration of strontium in keratin is low, so analysis requires large samples of fur and feathers (Appendix B, Table A3) [122,123]. Also, sample preparation and analyses are time and technique intensive [121,123,124]. Finally, migratory origin analysis using strontium may require the development of a unique bioavailable ^87^Sr/^86^Sr isoscape, but see [114]. Therefore, only a handful of studies have used strontium isotopes to track avian migration, with fewer studies in recent years [119,120,125,126]. We know of one study that used strontium isotope techniques to investigate the movements of migratory bats. Kruszynski et al. [39] coupled δ^2^H and ^87^Sr/^86^Sr to infer migratory pathways of *Pipistrellus nathusii* in Europe. Using δ^2^H, this study successfully identified movement pathways across Europe, but the combined use of δ^2^H and ^87^Sr/^86^Sr warrants further investigation in the context of bat migration, as there was not agreement between the probable origin maps for these two isotopic systems [39].

There is little to no strontium discrimination across trophic levels [127], so strontium isoscapes are not always constructed using the study species, e.g., [128,129,130,131]. However, Kruszynski et al. [39] reported a discrimination factor of 0.0028 ± 0.0002 between bioavailable ^87^Sr/^86^Sr and ^87^Sr/^86^Sr in the fur of *P. nathusii* and suggested further analysis of keratin structures in modern mammals to investigate a possible trophic discrimination factor between biologically available ^87^Sr/^86^Sr, and the ^87^Sr/^86^Sr signature in mammal fur. Therefore, a first step in further applying strontium isotope techniques to bat migratory systems is the generation of strontium isoscapes for the species and area of interest using samples taken from known-origin individuals during their summer residency, e.g., [28], or sedentary bat species occupying a similar niche to the migratory species of interest, e.g., [40]. Future research by bat biologists may focus on regions of the world with extensively developed strontium isoscapes, e.g., Europe and North America, or regions with large variation in bedrock type and age, e.g., Alaska and Spain. Additionally, strontium isoscapes may be particularly useful for recreating migratory pathways or demonstrating natal philopatry using tissues with differing turnover rates (studies involving teeth, bones, and fur are most promising). 

Migratory studies of modern taxa using strontium isotope techniques frequently do so in combination with one or more stable light isotopes, but see [126,132]. The most common second isotope is δ^18^O or δ^2^H, e.g., [39,47,76,120], but δ^13^C [119], δ^15^N [133], and/or δ^34^S may be included. Using multiple isotopes, a number of studies have successfully differentiated between local and non-local mammals within a predetermined area of interest, e.g., [77,134,135]. Fewer studies have sought to identify origin or piece together pathways of migratory taxa, e.g., [39,47,117,136]. Others have used patterns in ^87^Sr/^86^Sr to pose questions about behaviors, including natal dispersal and philopatry [137], niche occupancy [138], dietary calcium sources [125], and changes in migratory behavior over time [133]. Many of these applications are highly relevant to bat migratory systems. Specifically, the distinction between local and non-local is a useful one in studies of bats at congregation sites such as hibernacula, swarming sites, or large roosts.

The use of strontium isotope techniques includes several important analytical considerations. Due to the relatively low concentration of ^87^Sr/^86^Sr in keratin and the lesser understood method for incorporation, the initial method development may be required for analysis of keratinous tissues. There is a good foundation of literature to build on for this work, i.e., [123,124,139]. When live individuals are sampled, thermal ionization mass spectrometry (TIMS) may be the preferred analysis mechanism, as it relies on a smaller sample size than the more traditional multi collector inductively coupled plasma mass spectrometry method (MC-ICP-MS; Appendix B, Table A3). 

Proper preparation of biological samples is necessary to ensure the ^87^Sr/^86^Sr signature recorded after analysis is reflective of the ^87^Sr/^86^Sr signature of the tissue of interest during the time of formation. Feather and fur samples may contain exogenous (“superficial”) strontium, which is not incorporated into the internal keratin structure, and should be removed before analysis. Exogenous strontium likely originates from atmospheric or lithospheric strontium (i.e., soil and dust particles) as opposed to dietary strontium (i.e., food and water) [123,124]. In studies of bat migration, the removal of exogenous strontium is particularly important when analyzing unknown-origin fur collected outside of the summer residency period. In these cases, the location where the bat was captured may be distinct from the location where the fur was formed, and there is potential for exogenous strontium to contaminate the endogenous signal, contributing to additional noise in the ^87^Sr/^86^Sr signature, as seen in [39]. This extrinsic signature can be problematic but may also provide an opportunity, e.g., [124]. Future studies of bat migration may investigate whether the extrinsic signature could provide valuable land-use information about time periods when fur is not growing (e.g., hibernation) and may help identify the general location of important roost structures or hibernacula.

### 2.5. Using Trace Elements and Contaminants to Study Bat Migration

Trace elements and contaminants are used extensively to study migration in birds but have received little use by bat biologists for the same purpose, but see [38]. Trace elements may be referred to as trace metals, but the terms are not interchangeable; trace elements can refer to both metals and metalloids found at low concentrations (0.1%) in the earth’s crust while trace metals should only refer to rare cations [140]. Both are believed to be naturally incorporated into the biosphere via soil and water, and artificially via pollution [140]. Typically, trace elements present in the landscape via pollution or other anthropogenic activities are referred to as contaminants. However, the term contaminants can also be used to refer to organic pollutants or pesticides (e.g., organochlorides, dichlorodiphenyltrichloroethane, dichlorodiphenyldichloroethylene, polybrominated diphenyl ethers). 

Some studies were successful in mapping the distribution of contaminants across the landscape, e.g., [141]. Other studies have paired contaminant analyses with stable isotopes to address questions of contaminant exposure in avian systems (e.g., δ^34^S, δ^13^C, δ^15^N, δ^2^H, and Hg in *Phalacrocorax auratus* [142]). Contaminants have most commonly been used to track animal dispersal or migration by studying movements to and from highly contaminated areas, such as environmental contamination sites (e.g., heavy metal contaminant exposure at the Savannah River Site [143]), the Arctic (i.e., persistent organic pollutant bioaccumulation via atmospheric transport and deposition), e.g., [144,145,146], and some parts of Asia (e.g., polychlorinated biphenyl (PCB) exposure in southern Asia [147]). This is achieved by linking contaminant bioaccumulation to specific areas, e.g., [144,145,147], and pairing contaminant analyses with stable isotope techniques, e.g., [142]. While contaminants have been more thoroughly explored by bat biologists than trace elements [148], they have only been investigated under the lens of toxicity and contaminant exposure (likely via diet and drinking water), e.g., [149,150,151,152,153,154,155]. To our knowledge, contaminants have not yet been used to study migratory bat behavior or assign probable origin. Existing studies that demonstrate bioaccumulation of contaminants in bat tissues at sites near point sources of contaminants (e.g., chemical plants [151], urban centers [152], mines [155]) provide a framework that could be used in the future to track bat movements to and from these sites (e.g., questions of fidelity to maternity colonies or hibernacula). Of the potential contaminants, atmospheric mercury (Hg) is a promising place to start for studies of bat migration; it is correlated with mercury in the fur of some bat species (i.e., *Myotis lucifugus*, *M. septentrionalis*, *E. fuscus*), and its distribution can be mapped across the landscape [141], but see [38]. 

Trace element concentrations do not reliably vary at the landscape scale (i.e., with latitude, longitude, elevation), but see [38], making large-scale origin assignments impractical. Nonetheless, there can be substantial variation in trace element concentrations at relatively small scales. The development of more efficient extraction and analytical techniques in recent decades has allowed researchers to quantify the concentrations of many trace elements in small samples (Appendix B, Table A4; e.g., Donovan et al. [156] measured 62 trace elements in each 2 mg feather sample using ICP-MS techniques). The results of this approach can subsequently be narrowed down into “predictor elements”, or the elements that show enough variation to discriminate among the different groups of samples (usually achieved using a principal component or discriminant analysis). The predictor elements often depend on the study area, but magnesium (Mg) is often a common predictor [38,156,157,158,159,160,161,162,163]. Previous research using this technique to track bird migrations has shown that trace element profiles can differentiate among sites that are less than 4 km apart [157,159]. While these studies are unable to identify migratory origin across a large geographic landscape, they can pinpoint previously identified habitat, or assign origin across small landscapes, e.g., [157,159,162,163]. Bat-specific applications of trace element analyses may include making assignments on a regional or local scale, for example, when differentiating among breeding colonies [158]. 

In a recent and innovative development, Wieringa et al. [38] created a distribution map using 14 trace elements in soils across eastern North America, a much larger area than has previously been used to study migratory movements. They sampled fur from museum specimens of *Lasiurus borealis* to create a base map of the distribution of trace elements across the landscape and to assign known-origin bats to locations of origin based on the trace element profiles in their fur [38]. The study showed ~80% accuracy in the training dataset with 50% precision [38]. Wieringa et al. [38] emphasized accuracy over precision, and the results were less precise and accurate when compared to studies using stable light isotopes (especially when compared with δ^2^H [164]). Future research by bat biologists could expand on the methods established by Wieringa et al. [38] to map trace element distribution across the landscape, as well as improve the accuracy and precision of origin assignments using this method. Additionally, researchers could expand the use of these methods to migratory systems in regions outside of North America, bearing in mind that it is best practice to characterize the abiotic (e.g., soil) trace element distribution across the landscape before expanding to biotic systems (e.g., bats). Researchers should also consider pairing this technique with more broadly understood intrinsic marking techniques (e.g., δ^2^H; see Section 2.6).

### 2.6. Using Paired Techniques to Study Bat Migration

Although intrinsic marking techniques have many benefits, their biggest drawback is the low resolution at which origin assignments can be made. For species that commonly migrate large distances, and for questions addressing minimum distance traveled, assignment resolution may not hinder the research objectives, e.g., [20,28]. However, for species moving regionally, e.g., [64,68], across longitudes, e.g., [120], or in habitats with high homogeneity of the marker of interest, e.g., [30], the degree of specificity in origin assignments may contribute to the success of the study. Additionally, as studies of bat migration become more commonplace, complex questions (e.g., those addressing both migratory movements and dietary needs) may also become more common, e.g., [90,108]. In these instances, using multiple intrinsic marking techniques or paired intrinsic and extrinsic marking techniques may be the most appropriate approach.

The use of multiple isotopes to identify migratory origin can improve both accuracy and precision of assignment. Popa-Lisseanu et al. [40] used three stable light isotopes (δ^2^H, δ^13^C, and δ^15^N) to identify probable origin locations of migratory bats in Europe and found the accuracy of assignments increased from 47.4% (using only δ^2^H), to 86–89.5% (using δ^2^H/δ^15^N and δ^2^H/δ^13^C, respectively), to 93% (using all three) [40]. Bataille et al. [165] used ^87^Sr/^86^Sr, δ^34^S, and δ^18^O to assign probable origin of canine teeth from archaeological remains in Brittany, France. The researchers showed increasingly precise assignments with two and three isotopes when compared with ^87^Sr/^86^Sr, δ^34^S, or δ^18^O alone [165]. While these studies incorporated paired stable and radiogenic isotope techniques, there are many more intrinsic marking techniques to consider. Migratory bird studies have incorporated trace elements, e.g., [166,167], contaminants, e.g., [142,168], genetics, e.g., [31,95], song dialects, e.g., [169], phenotypic characteristics (“biometrics”), e.g., [158], flight direction, e.g., [170], and a priori knowledge about the study species, e.g., [171] to investigate migratory pathways or seasonal and/or natal origin, in addition to population structure and habitat use. While some of these (i.e., genetics) are beyond the scope of this review, they are helpful tools to consider for certain research objectives. 

Although most multi-isotope studies of bat migration use some combination of δ^2^H, δ^13^C, and/or δ^15^N e.g., [30,40,61,65], recently, researchers have begun to incorporate δ^34^S, ^87^Sr/^86^Sr, and trace elements [38,39,68] into studies of bat migration. Additional information incorporated by bat biologists via a priori knowledge has included species range [63]; niche occupancy [41]; density based on museum records [19]; preferred elevation [63]; previous records of dispersal distance [172]; and previous migratory flight bearings [32]. Dietary preference is likely also important; for example, the stable hydrogen isotope composition of aquatic insects are distinct from sympatric terrestrial insects [41,172]. Ultimately, the most appropriate pairings of intrinsic markers will depend on the biology and behavior of the study species, the heterogeneity of the landscape, and the research question. It is clear that a deep knowledge of study species biology can improve the accuracy and precision of origin assignments based on intrinsic markers.

Recent advances in Passive Integrated Transponder (PIT) tag and radio/satellite transmitter technology have reinvigorated the use of extrinsic markers to study bat migration, e.g., [173,174]. These advances have expanded the available techniques that may be paired with intrinsic markers to study migratory behavior. Many studies have paired extrinsic and intrinsic marking techniques to study migratory birds, and this is an approach that holds much promise in bat biology. The most common combination of intrinsic and extrinsic markers is stable light isotopes and band recovery data, e.g., [175,176,177], but see [63]. By combining these techniques, researchers can infer both migratory pathway and origin, while also decreasing the bias associated with using band recovery data alone [178]. Despite the promise of this combination, the previously documented low recapture rates for marked bats and decreased survivability associated with banding of certain bat species may hinder the widespread feasibility of these coupled techniques for bat biologists [9,24,179]. Recently, however, researchers have developed passive detection mechanisms for PIT tags that result in increased recapture rates [180] without implicating flight behavior of otherwise affected species [181]. Another important and newly developed mechanism for inferring migratory direction is the circular release box for bats (CRBox), which allows inferences to be made about orientation behavior and flight direction [182,183]. Additionally, the Motus Wildlife Tracking System (“MOTUS”) was established on a continent-wide scale in 2014 to passively track radio-tagged aerial organisms (i.e., birds, bats, insects) via remote receiver stations [25]. Since its introduction, several species of bats have been tracked using concepts employed by the Motus network e.g., [26,27,184]. Future directions for biologists interested in using multiple techniques to study bat migration are vast. In studies of swarming and hibernating species, researchers could use a combination of intrinsic markers and PIT tag readers to address both long term (since summer fur replacement) and short-term (among swarming site) movements of bat species. The combination of intrinsic marker techniques and the MOTUS network or CRBox during fall movements of long-distance migratory bat species may allow researchers to both identify migratory origin and track future migratory distance and direction. Finally, the combination of flight direction with isotopic analyses could improve origin assignment precision, especially if combined with extrinsic techniques (i.e., radio/satellite tracking, PIT tags) and/or MOTUS. 

## 3. Tissue Selection for Intrinsic Marker Analysis in Bats

Intrinsic marker analyses may be conducted on various tissue types, and the selection of the most suitable tissue(s) is a critical step in any study [37]. Important factors include whether the tissue type is metabolically active or inert; the period in the animal’s life that is reflected by the chosen tissue (related to tissue turnover rate and the timing of growth); the quantity of the marker of interest within the tissue (depending on the sample mass required for analysis); and the invasiveness of sampling different tissue types [41]. In bats, some common tissues can be sampled non-lethally for intrinsic marker analysis (e.g., wing membrane [185], blood [186], fur [43] and claw [70]) while the processes for sampling others are highly invasive or lethal (e.g., liver, muscle, and bone collagen) [17,84,187].

**Figure 1:**
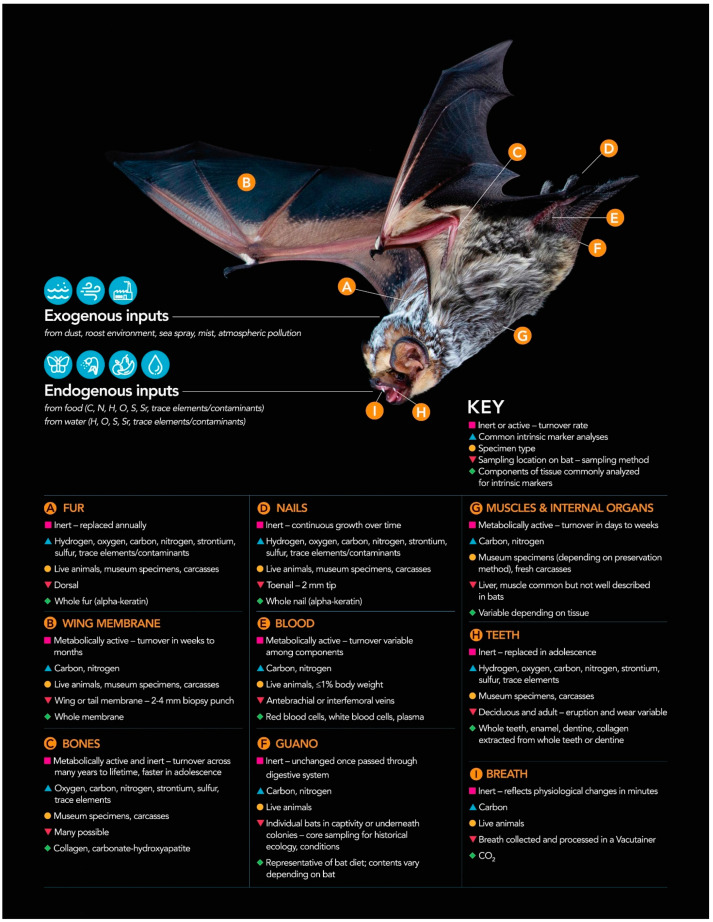
Summary of bat tissue sampling practices for intrinsic marker analyses: Intrinsic markers are present and may be quantified following sampling of multiple tissues in this hoary bat (*Lasiurus cinereus*). Sources of intrinsic markers include both endogenous and exogenous inputs. Tissues may be inert or active (with varying tissue turnover rates); some may be sampled from dead bats only and other from live bats with varying levels of invasiveness. Photo credit: Sherri Fenton and M. Brock Fenton; graphic design: Lori Lee Pike.

Most tissues are either metabolically inert or active. Metabolically active tissues continually regenerate and thus have a chemical composition that is continuously changing. Examples include the blood, muscles, liver, and wing membrane [187,188,189]. Comparatively, metabolically inert tissues are fixed after formation and are reflective of the conditions during that development period (e.g., fur) [187,188,189]. Breath, while not a tissue, is frequently sampled for intrinsic marker analysis and shares salient characteristics with tissues, so will also be discussed here [42,190,191]. Figure 1 presents a graphical summary of the tissue types that may be used for intrinsic marker investigations of bat ecology.

### 3.1. Metabolically Inert Tissues in Bats

In studies using intrinsic markers to investigate bat migration, fur is the dominant tissue type used. Bat fur is usually assumed to be replaced annually through molting. The typical molting pattern in temperate bats is an annual molt during the summer–fall before migration, but factors such as sex, age or migratory behavior may contribute to some bats molting outside of the usual timeframe [28,192] For example, molting may be postponed until after lactation or disrupted during pregnancy, reproduction, or other energy demanding processes and will vary with age and sex [28,192]. Typically, fur samples are taken from the upper dorsal region between the shoulder blades [43,188]; growth may be asynchronous between ventral and dorsal surfaces [28]. Understanding the molting cycles helps to further the accuracy and predictability of fur isotopic composition and to gain a deeper understanding of bat behavior.

Other metabolically inert tissues are frequently used in studies of other taxa, but have received little attention in modern bats, include teeth and bone collagen [43,188]. Bone collagen is developed early in life [189] and is metabolically active initially but with age the turnover rate slows to a negligible rate. The teeth consist of: (1) enamel, a hard outer layer, (2) primary dentine, an inner layer beneath enamel, both formed during infancy above the gum line; (3) secondary dentine, which continually forms new layers, and (4) cementum, an outer layer, both continuously formed at the root of the tooth below the gum line [193]. Many bats have deciduous teeth which they shed at variable frequencies during their infancy and are replaced with their adult teeth [194]. Both tooth and bone collagen could provide information about individual bats when they were juveniles or subadults because these tissues are active during their growth and inert when formed.

Inert tissues that grow continuously over longer time periods present an opportunity for time series analyses. For example, in some mammals, individual hair strands can be sampled at varying locations along their length to gain information about the animal at various points in its lifetime [195,196,197,198]. We do not know of any bats that have continuously growing fur, but the hind claws of bats may potentially be sampled at varying lengths to achieve the same goal. There is little information about the growth patterns and timing of bat claws, but Ethier et al. [199] provide a useful summary of patterns in mammalian claw growth. To date, claw tissue has been infrequently used to study bats, likely because of their size and the invasiveness of claw removal, but see [70,200].

### 3.2. Turnover of Metabolically Active Tissues in Bats

The rate at which a chemical marker in a metabolically active tissue is replaced by the same marker from another source is known as a tissue turnover rate. The timing of tissue growth and turnover is critical information, as these factors inform the time period about which markers are providing information. Turnover rates may range from minutes to years [187]. Tissue turnover is often quantified as the half-life (t_50_) of the marker of interest, i.e., when turnover occurs in half of the markers in the tissue [189]. The turnover rate of an intrinsic chemical marker varies among tissues, and among different markers within the same tissue. This latter variation occurs because of metabolic decoupling, change in diet/nutrients, or variation in nutrient routing (e.g., carbon sourced from protein or carbohydrate) [201]. In most cases, research on the turnover rates of metabolically active tissues has focused on the turnover rates of carbon and nitrogen, because of predictable discrimination factors of ~0.2‰ and 2.2–3.4‰ between trophic levels, respectively [43,87,188], and the turnover rates of other intrinsic markers are a substantial knowledge gap. 

Diet-switching studies on captive animals provide most of the information on turnover rates [190,201]. Less commonly, in wild populations, variation over time in the intrinsic marker composition of metabolically active tissues can be used to infer turnover rate [42,90]. There have been several studies that have explicitly investigated tissue turnover rates in bats [43,188,191,201]. In our summary below, we will report bat-specific findings where possible, and findings from other taxa when not. A more complete summary is included in Table 1. 

The turnover rate of carbon in CO_2_ in breath is widely studied because breath samples can provide information on very recent (minutes to hours) dietary patterns, with t_50_ turnover rates of 27.3 ± 6.4 min in *Noctilio albiventris* [191], 18.6 min in *Desmodus rotundus* [210], and 10.9 ± 7.5 min in *Carollia perspicillata* [190]. Diet switching experiments show that the variance occurring in breath turnover rates is likely due to the different ratios of proteins and sugars consumed [190]. We are unaware of reported liver turnover rates in bats, but Tieszen et al. [187] recorded the t_50_ of carbon in *Meriones* *unguiculatus* (referred to as *M.* *unguienlatus*) liver to be 6.4 days. Depending on which components are used, the turnover rate of carbon and nitrogen in blood varies. Reported values include 24 to 39 days for *Glossophaga soricina* (whole blood cells) [201], 120 to 126 days for *G. soricina* and *L. curasoae* (whole blood cells) [43], 2.9 days (plasma), and 29.8 days (cellular) for *Corvus brachyrhynchos* [211]. The t_50_ of carbon in *M.* *unguiculatus* muscle tissue is 27.6 days [187].

Wing membrane is a tissue that is unique to bats, and one that is commonly sampled in a relatively minimally invasive way using a biopsy punch. Following sampling, reports of wing membrane regeneration include 3 to 4 weeks in *L. curasoae* and *G. soricina* [43] and 27.3 ± 12.2 days (wing membrane) or 18.3 ± 4.3 days (tail membrane) in *E. fuscus* [185]. Voigt et al. [43] suggest that biochemical processes help the wing tissue regenerate following injury, resulting in regrowth faster than the actual turnover rate of the tissue. They report a t_50_ of carbon in wing membrane to be between 102–134 days and suggest that the low turnover rate of wing membrane could be due to high concentrations of bone collagen found within the wing membrane. Roswag et al. [188] observed the wing membrane turnover rate of *N. noctula* to be 7 weeks. Although most data on wing membrane turnover rates come from laboratory studies, Frick et al. [42] documented seasonal (winter to spring) turnover rates in the wing membrane of *Antrozous pallidus*.

Turnover rates of specific intrinsic markers in the same tissue type may vary substantially, likely related to variation in metabolic rate associated with diet change or changes in energetic requirements (e.g., during migration) [211,212]. Bats eating a diet with a lower C:N ratio had a slower carbon turnover rate in blood than those consuming a diet with high C:N, while the nitrogen turnover rate remained similar [201]. During periods with high energy requirements, bats may increase both food consumption and metabolic rate with corresponding shifts in nutrient routing [42,90,203,209]. The seasonal availability of certain foods may cause changes in nutrient routing, with some intrinsic markers being immediately metabolized, while others are incorporated into new tissues [42,43,190].

The effects of torpor on the incorporation of intrinsic markers into tissues, and the tissues’ turnover rates, are unknown. However, frequent use of torpor by bats undoubtedly plays an important role in tissue turnover rates. Torpor alters the metabolic rate of bats allowing for the conservation of energy, especially during cold periods or periods when endogenous energy stores are low [18]. Because metabolic activity has a direct relationship with tissue turnover rate [211], the tissues of torpid bats would be expected to turnover more slowly than non-torpid bats. Males and non-reproductive females enter torpor more frequently than reproductive females [18] potentially leading to intraspecific variation in tissue turnover rates.

### 3.3. Discrimination Factors

Variation in diet may also result in variation in diet-tissue discrimination, which has been best illustrated using stable isotopes of carbon and nitrogen but is likely relevant for other markers. The carbohydrates within plant-based foods are typically metabolized quickly and the resulting CO_2_ is exhaled, while the small amount of protein within the plant is used in tissue catabolism [190]. In omnivorous bats, carbon in the wing membrane mainly originates from protein in the insect portion of the diet while the carbon in breath originates from carbohydrates in fruit [42,190]. As a result of their high protein diet, insectivorous bats often have higher δ^13^C_tissue_ than nectarivorous or frugivorous bats, but similar δ^13^C_breath_. Therefore, there is a direct relationship between the trophic level and the difference between breath δ^13^C and tissue δ^13^C [190]. Turnover rates of δ^15^N can vary with dietary source, as protein can be sourced both externally, via diet, and internally through the nitrogen cycle [203,213]. Internal nitrogen is enriched because it has been previously metabolized [203]. The nitrogen cycle has several reservoirs of nitrogen, and other biological processes, such as pancreas secretion, that can contribute to endogenous nitrogen sources [213]. 

### 3.4. Approaches to Tissue Sampling

The mass of each sample needed for isotopic analysis is a critical consideration because the small size of most bats limits the quantity of tissues that can be sampled non-lethally. The key considerations are the amount of the marker of interest in the tissue; the sensitivity of the laboratory equipment to detect the marker of interest; and the quantity of tissue that may be taken from an individual bat. Table 1 and Appendix B summarize sample masses that have been used for various intrinsic marker analyses of a range of tissues. 

A small amount of literature exists discussing specific practices for sampling blood, wing membrane, and fur from bats. The sampling of blood has been particularly evaluated; Baer and McLean [214] originally suggested the removal of 0.1–0.2 mL of blood from the jugular vein of small bats (in this case *Tadarida brasiliensis*), although more recent studies have suggested an order of magnitude smaller. Wimsatt et al. [215] sampled 58 ± 12 µL from the interfemoral vein in *E. fuscus* under anesthesia without impacting survivability. Smith et al. [216] sampled blood from the brachial and propatagial veins in eight species of microbats and suggested 6 µL/g of body mass. This study was quickly refuted by Racey et al. [217] who suggested sampling from the interfemoral vein to avoid impacting flight. Eshar and Weinberg [186] suggested the removal of blood ≤ 1% of total body weight from either the interfemoral or cephalic vein (providing detailed instructions for sampling blood in bats, using *Rousettus aegyptiacus* as an example). The sampling of wing membrane has been evaluated to a lesser extent; both Faure et al. [185] and Pollock et al. [218] studied propatagium sampling techniques in *E. fuscus* and suggest sampling tail membrane tissue over wing membrane tissue; the increased vasculature in tail membrane causes wounds in the tail to heal significantly faster compared to the wing. Finally, Fraser et al. [192] details considerations when sampling fur from various bat species, accounting for differences in molting patterns and timelines.

## 4. Overview of Workflow

**Figure 2:**
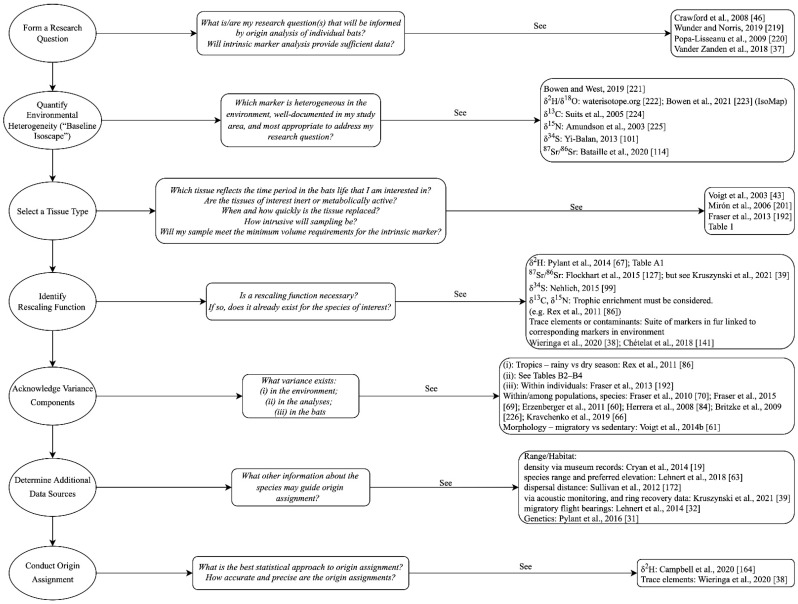
A generalized workflow for conception and implementation of a study using intrinsic markers to track migratory bat species. The far-right column lists bat-specific literature (except where more general literature is appropriate) that specifically addresses each stage [19,31,32,37,38,39,43,46,60,61,63,66,67,69,70,84,86,99,101,114,141,164,172,192,201,219,220,221,222,223,224,225,226]. Notice the extensive knowledge gaps that exist for the lesser used markers, especially in the last step “conduct origin assignment”. Steps in far-left column modified from Vander Zanden et al. [37].

While some intrinsic markers (e.g., stable hydrogen isotopes) have been used extensively to study bat migration, others are in their infancy for this purpose. Because intrinsic marker analyses of tissues provide indirect evidence of bat movement, the use of any markers for migration research requires significant background knowledge, modelling, and assumptions. Vander Zanden et al. [37] presented a generalized workflow for designing a study to track animal movement using stable isotope analyses of tissue samples. In Figure 2, we present a modified version of this workflow that can be applied to any of the intrinsic markers discussed in the present paper. We provide important questions for consideration at each stage and a summary of existing bat-specific literature (where appropriate) that has explicitly addressed the methodological considerations associated with each step. This summary highlights the volume of work that has been conducted in this area, as well as the knowledge gaps. Aligned with the greater volume of work that has used stable hydrogen isotope techniques to study bat migration, there has been significant attention to the methods associated with this technique. Bat-specific rescaling functions and associated isoscapes for markers that have been used less frequently (e.g., strontium, sulfur) are less prevalent or entirely absent, but see [38,39], and intrinsic and extrinsic marker techniques have not been combined as frequently as in avian research. The majority of work has focused on analyses of fur, but there is great potential to analyze multiple tissues to learn about different time periods in an individual bat’s life. e.g., [42,43]. Conducting this work well requires further investigations of tissue growth and turnover rates, as well as laboratory work to modify and develop analytical techniques (e.g., stable hydrogen isotope analyses in non-keratinous tissues). 

## 5. Conclusions

There is close to a thirty-year history of using intrinsic markers to study bat migration [17] and in the past fifteen years, applications have particularly proliferated. The ability to make origin estimates of individual migratory bats has furthered our understanding of migratory patterns, as well as the migratory ecology of these elusive animals. Stable hydrogen isotope techniques have been the leading approach, but marker choice is dependent on both the research question and the study area. Recent innovations in analytical techniques have made lesser used intrinsic markers (e.g., trace elements/contaminants, strontium) and the combined analysis of intrinsic markers increasingly accessible and informative, although logistical challenges still exist. There is clearly much important methodological innovation to be achieved in the applications of these lesser used intrinsic markers in making inferences about bat movements, especially if the goal is to estimate probabilities of origin. Combinations of intrinsic marker analyses can be particularly powerful in estimating migratory origin and, even in the absence of clearly defined isoscapes, can allow researchers to address simple but important questions about whether congregating groups of bats consist of individuals from few or many locations, e.g., [64]. As extrinsic marking technologies advance and become more accessible to bat research, there is further potential to combine these with intrinsic marking techniques.

## Figures and Tables

**Table 1:** Turnover rate and sample mass reported for the most common tissues used in intrinsic marker analyses. Also included is the mean reported weight of various bat species; when available, this was obtained from the original literature source. Otherwise, an additional reference was included to quantify body mass of the study species (in these cases, the original literature source is always reported first, while the additional source for body mass is reported second). Information denoted with an asterisk (*) was sourced from Gashchak et al. [202]. Information denoted with a double asterisk (**) was sourced from Voigt et al. [43]. Turnover rate key: m = minutes; d = days; w = weeks; mo = months. All turnover rates reflect carbon except where otherwise indicated.

Tissue Type	Turnover Rate	Amount of Tissue	Study Species	Mass of Species (g)	Reference
Wing Membrane		3 mm biopsy	*A. pallidus*	15.8	[42,203]
		2 mm biopsy, (≥0.1 mg)	*Myotis daubentonii*, *N. noctula*, *Nyctalus leiseri*	*N.noctula*: $\bar{x}$ = 27.7 *	[204]
	δ^13^C & δ^15^N: 7 w	3.5 mm biopsy	*N. noctula*	$\bar{x}$ = 27.7 *	[188]
	t_50_ = 102−134 d	3 mm biopsy (2/wing)	*L. curasoae*, *G. soricina*	*L. curasoae*: 23.6 ± 2.1 *G. soricina*: 10.2 ± 0.7	[43]
Bone Collagen	Low (yearly to lifetime)		*L. curasoae*, *G. soricina*	*L. curasoae*: 23.6 ± 2.1 *G. soricina*: 10.2 ± 0.7	[43]
	Early life: rapid; late life: 500 d-life		*Rattus* spp.	235	[189]
Muscle	δ^15^N: 6–8 w	12–15 mg	*Rousettus aegyptiacus*	110–160	[84,205]
	t_50_ = 27.6 d	5–10 mg	*M.unguiculatus* (gerbil)	67.7 ± 6.0	[187,206]
Liver	t_50_ = 6.4 d, t_99.99_ = 84 d	5–10 mg	*M. unguiculatus* (gerbil)	67.7 ± 6.0	[187,206]
Blood	δ^13^C & δ^15^N: 24–39 d		*G. soricina*	10.2 ± 0.7 **	[201]
		0.5–1 mg	*Nyctalus lasiopterus*	41–76	[207,208]
		50 µL	*A. pallidus*	15.8	[20,203]
	t_50_ = 120–126 d	30 µL (propatagial vein)	*L. curasoae*, *G. soricina*	*L. curasoae*: 23.6 ± 2.1 *G. soricina*: 10.2 ± 0.7	[43]
Breath CO_2_	t_50_ = 27.3 ± 6.4 m	10 mL	*Noctilio albiventris*	22.1 ± 3.1 (fasted); 27.3 ± 2.9 (foraging)	[191]
		Sample accumulated for 1.5 m	*Pipistrellus nathusii*	7.6 ± 0.6	[209]
	t_50_ = 9.5 ± 6.1 m (Hexose) t_50_ = 9.5 ± 7.0 m (Fructose) t_50_ = 13.8 ± 9.4 m (Protein/Fructose) $\bar{x}$= 10.9 ± 7.5 m (all diets)		*Carollia perspicillata*	$\bar{x}$ = 21.76	[190]
		18 mL	*L. noctivagans*	11.30 ± 1.45	[90]
		Sample accumulated for 5 m	*A. pallidus*	15.8	[42,203]
	18.6 m	3 m/10 mL	*Desmodus rotundus*	30.4 ± 3.2	[210]
Fur	δ^13^C & δ^15^N: >7 w	Upper tips (dorsal)	*N. noctula*	$\bar{x}$ = 27.7 *	[188]
	t_50_ mean = 537 d	0.25 cm^2^ (dorsal)	*L. curasoae*, *G. soricina*	*L. curasoae*: 23.6 ± 2.1 *G. soricina*: 10.2 ± 0.7	[43]

## Data Availability

No new data were created or analyzed in this study. Data sharing is not applicable to this article.

## Appendix A. Summary of Current Bat Literature Which Provide Rescaling Function Equations for δ^2^H, δ^13^C, and δ^15^N

**Table:**

Study Species	Common Name	Migratory Status	Intrinsic Marker	Region	Regression Method	Precipitation	Gender	Equation	*n*	r^2^	*p*-Value	Reference
*Barbastella barbastellus*	Western barbastelle	sedentary	δ^2^H	Europe	RMA	Mean annual precipitation	combined	δ^2^H_f_ = (1.37 × δ^2^H_p_) − 5.52	217	0.67	<0.001	[15]
*Barbastella barbastellus*	Western barbastelle	sedentary	δ^2^H	Europe	LMM / REML	Mean annual precipitation	combined	δ^2^H_f_ = (1.07 × δ^2^H_map_) − 16.84	178	0.72	NR*	[40]
*Chaerephon cf. pumilus*	Little free-tailed bat	sedentary	δ^15^N	Africa	OLS	N/A*	combined	^15^N_f_ = (−0.01 × elevation) + 28.78	55	0.32	<0.001	[61]
*Eidolon helvum*	Straw-colored fruit bat	migratory	δ^2^H	Africa	RMA	Mean annual precipitation	combined	δ^2^H_f_ = (1.52 × δ^2^H_p_) − 54.09	193	NR*	<0.001	[30]
*Epomophorus crypturus*	Peters’s epauletted fruit bat	likely non-migratory	δ^2^H	Africa	RMA	Mean annual precipitation	combined	δ^2^H_f_ = (1.5 2 × δ^2^H_p_) − 54.09	193	NR*	<0.001	[30]
*Epomophorus wahlbergi*	Wahlberg’s epauletted fruit bat	likely non-migratory	δ^2^H	Africa	RMA	Mean annual precipitation	combined	δ^2^H_f_ = (1.52 × δ^2^H_p_) − 54.09	193	NR*	<0.001	[30]
*Epomophorus wahlbergi*	Wahlberg’s epauletted fruit bat	likely non-migratory	δ^13^C, δ^15^N	Africa	OLS	N/A*	combined	elevation = 4635 − (67 × ^15^N_f_) + (112 × ^13^C_f_)	66, 65	0.22	0.004, 0.002	[61]
*Epomops franqueti*	Franquet’s epauletted fruit bat	likely non-migratory	δ^2^H	Africa	RMA	Mean annual precipitation	combined	δ^2^H_f_ = (1.52 × δ^2^H_p_) − 54.09	193	NR*	<0.001	[30]
*Eptesicus isabellinus*	Meridional serotine	sedentary	δ^2^H	Europe	RMA	Mean annual precipitation	combined	δ^2^H_f_ = (1.37 × δ^2^H_p_) − 5.52	217	0.67	<0.001	[15]
*Eptesicus isabellinus*	Meridional serotine	sedentary	δ^2^H	Europe	LMM / REML	Mean annual precipitation	combined	δ^2^H_f_ = (1.07 × δ^2^H_map_) − 16.84	178	0.72	NR*	[40]
*Eptesicus serotinus*	Serotine bat	sedentary	δ^2^H	Europe	RMA	Mean annual precipitation	combined	δ^2^H_f_ = (1.37 × δ^2^H_p_) − 5.52	217	0.67	<0.001	[15]
*Eptesicus serotinus*	Serotine bat	sedentary	δ^2^H	Europe	LMM / REML	Mean annual precipitation	combined	δ^2^H_f_ = (1.07 × δ^2^H_map_) − 16.84	178	0.72	NR*	[40]
*Hipposideros caffer*	Sundevall’s roundleaf bat	sedentary	δ^15^N	Africa	OLS	N/A*	combined	^15^N_f_ = (−0.01 × elevation) + 28.78	55	0.32	<0.001	[61]
*Hypsignathus monstrosus*	Hammer-headed bat	likely non-migratory	δ^2^H	Africa	RMA	Mean annual precipitation	combined	δ^2^H_f_ = (1.52 × δ^2^H_p_) − 54.09	193	NR*	<0.001	[30]
*Lasionycteris noctivagans*	Silver-haired bat	migratory	δ^2^H	North America	geostatistical model	Mean growing season precipitation	combined	δ^2^H_f_ = (0.70 × δ^2^H_p_) − 40.65	NR*	0.67	<0.001	[20]
*Lasiurus borealis*	Eastern red bat	migratory	δ^2^H	North America	GLM	Mean annual precipitation	male	δ^2^H_f_ = (−0.82 × δ^2^H_p_) − 58.80	17	0.33	0.0482	[226]
							female	δ^2^H_f_ = (1.35 × δ^2^H_p_) − 6.30	36	0.31	0.0003	
							juvenile	δ^2^H_f_ = (0.67 × δ^2^H_p_) − 23.97	28	0.16	0.0143	
							combined	δ^2^H_f_ = (0.48 × δ^2^H_p_) − 26.10	81	0.07	0.0201	
*Lasiurus borealis*	Eastern red bat	migratory	δ^2^H	North America	RMA	Mean growing season precipitation	male	δ^2^H_f_ = (1.48 × δ^2^H_p_) + 13.95	20	0.69	<0.001	[67]
							female	δ^2^H_f_ = (1.75 × δ^2^H_p_) + 18.02	44	0.29	<0.001	
							combined	δ^2^H_f_ = (1.67 × δ^2^H_p_) + 16.84	64	0.37	<0.001	
*Lasiurus borealis*	Eastern red bat	migratory	δ^2^H	North America	RMA	Mean annual precipitation	combined	δ^2^H_f_ = (1.00 × δ^2^H_p_) + 8.17	64	0.41	<0.001	[31]
*Lasiurus cinereus*	Hoary bat	migratory	δ^2^H	North America	NR*	Mean growing season precipitation	combined	δ^2^H_f_ = (0.7884 × δ^2^H_p_) − 24.81	104	0.60	<0.001	[28]
*Lasiurus cinereus*	Hoary bat	migratory	δ^2^H	North America	OLS	Mean growing season precipitation	combined	δ^2^H_f_ = (0.73 × δ^2^H_p_) − 42.61	117	0.55	<0.001	[19]
*Lasiurus cinereus*	Hoary bat	migratory	δ^2^H	North America	RMA	Mean June/July/August precipitation	combined	δ^2^H_f_ = (0.874 × δ^2^H_p_) − 41.8	117	0.49	<0.001	[31]
*Lissonycteris angolensis*	Angolan fruit bat	sedentary	δ^2^H	Africa	RMA	Mean annual precipitation	combined	δ^2^H_f_ = (1.52 × δ^2^H_p_) − 54.09	193	NR*	<0.001	[30]
*Lissonycteris angolensis*	Angolan fruit bat	sedentary	δ^13^C, δ^15^N	Africa	OLS	N/A*	combined	elevation = 4635 − (67 × ^15^N_f_) + (112 * ^13^C_f_)	66, 65	0.22	0.004, 0.002	[61]
*Miniopterus natalensis*	Natal long-fingered bat	migratory	δ^15^N	Africa	OLS	N/A*	combined	^15^N_f_ = (−0.01 × elevation) + 28.78	55	0.32	<0.001	[61]
*Miniopterus schreibersii*	Schreiber’s bat	migratory	δ^2^H	Europe	LMM	growing season precipitation	combined	δ^2^H_f_ = (0.62 × δ^2^H_isoscape_) − 14.66	NR*	NR*	NR*	[227]
								δ^2^H_wing_ = (0.64 × δ^2^H_isoscape_) − 14.64	NR*	NR*	NR*	
*Myotis lucifugus*	Little brown myotis	migratory	δ^2^H	North America	GLM	Mean annual precipitation	male	δ^2^H_f_ = (0.49 × δ^2^H_p_) − 30.90	12	0.19	0.1527	[226]
							female	δ^2^H_f_ = (0.33 × δ^2^H_p_) − 40.41	54	0.06	0.0492	
							juvenile	δ^2^H_f_ = (1.09 × δ^2^H_p_) − 9.31	12	0.40	0.1291	
							combined	δ^2^H_f_ = (0.52 × δ^2^H_p_) − 30.82	78	0.17	0.0002	
*Myotis lucifugus*	Little brown myotis	migratory	δ^2^H	North America	OLS	Mean growing season precipitation	combined	δ^2^H_f_ = (2.69 × δ^2^H_p_) + 96.93	NR*	0.63	<0.001	[172]
*Myotis septentrionalis*	Northern myotis	migratory	δ^2^H	North America	GLM	Mean annual precipitation	male	δ^2^H_f_ = (0.79 × δ^2^H_p_) − 4.73	10	0.53	0.0088	[226]
							female	δ^2^H_f_ = (1.25 × δ^2^H_p_) + 18.48	16	0.71	0.0001	
							juvenile	δ^2^H_f_ = (1.65 × δ^2^H_p_) + 17.64	7	0.47	0.0258	
							combined	δ^2^H_f_ = (0.98 × δ^2^H_p_) + 5.48	33	0.54	<0.0001	
*Myotis sodalis*	Indiana bat	migratory	δ^2^H	North America	GLM	Mean annual precipitation	male	δ^2^H_f_ = (0.90 × δ^2^H_p_) − 0.59	12	0.46	0.0115	[226]
							female	δ^2^H_f_ = (0.71 × δ^2^H_p_) − 8.17	39	0.35	0.0001	
							juvenile	δ^2^H_f_ = (2.18 × δ^2^H_p_) + 30.33	8	0.63	0.0046	
							combined	δ^2^H_f_ = (0.83 × δ^2^H_p_) − 2.97	59	0.49	0.0001	
*Neoromicia nana*	Banana pipistrelle	sedentary	δ^15^N	Africa	OLS	N/A*	combined	^15^N_f_ = (−0.01 × elevation) + 28.78	55	0.32	<0.001	[61]
*Nyctalus leisleri*	Leisler’s bats	migratory	δ^2^H	Europe	RMA	Mean annual precipitation	combined	δ^2^H_f_ = (1.27 × δ^2^H_p_) − 7.35	178	NR*	<0.001	[34]
*Nyctalus noctula*	Common noctule	migratory	δ^2^H	Europe	RMA	Mean annual precipitation	combined	δ^2^H_f_ = (1.37 × δ^2^H_p_) − 5.52	217	0.67	<0.001	[15]
*Nyctalus noctula*	Common noctule	migratory	δ^2^H	Europe	RMA	Mean annual precipitation	combined	δ^2^H_f_ = (1.27 × δ^2^H_p_) − 7.35	178	NR*	<0.001	[34]
*Nyctalus noctula*	Common noctule	migratory	δ^2^H	Europe	LMM	Mean annual precipitation	combined	δ^2^H_f_ = (0.92 × δ^2^H_p_) − 30.72	335	NR*	NR*	[63]
*Nycteris thebaica*	Egyptian slit-faced bat	likely non-migratory	δ^15^N	Africa	OLS	N/A*	combined	^15^N_f_ = (−0.01 × elevation) + 28.78	55	0.32	<0.001	[61]
*Perimyotis subflavus*	Tri-colored bat	migratory	δ^2^H	North America	quadratic	Mean growing season precipitation	male	δ^2^H_f_ = (−0.036 × δ^2^H_p_^2^)-(1.789 × δ^2^H_p_) − 45.607	29	0.86	<0.01	[29]
							female	δ^2^H_f_ = (−0.034 × δ^2^H_p_^2^) − (1.606 × δ^2^H_p_) − 40.375	27	0.75	<0.01	
*Pipistrellus cf. grandidieri*	Dobson’s pipistrelle	sedentary	δ^15^N	Africa	OLS	N/A*	combined	^15^N_f_ = (−0.01 × elevation) + 28.78	55	0.32	<0.001	[61]
*Pipistrellus pipistrellus*	Common pipistrelles	sedentary?	δ^2^H	Europe	RMA	Mean annual precipitation	combined	δ^2^H_f_ = (1.27 × δ^2^H_p_) − 7.35	178	NR*	<0.001	[34]
*Pipistrellus nathusii*	Nathusius’ pipistrelles	migratory	δ^2^H	Europe	RMA	Mean annual precipitation	combined	δ^2^H_f_ = (1.27 × δ^2^H_p_) − 7.35	178	NR*	<0.001	[34]
*Pipistrellus nathusii*	Nathusius’ pipistrelles	migratory	δ^2^H	Europe	NR*	Mean annual precipitation	combined	δ^2^H_f_ = (0.74 × δ^2^H_p_) − 83.96	458	NR*	NR*	[39]
*Pipistrellus* sp.		sedentary	δ^15^N	Africa	OLS	N/A*	combined	^15^N_f_ = (−0.01 × elevation) + 28.78	55	0.32	<0.001	[61]
*Plecotus auritus*	Brown long-eared bat	sedentary	δ^2^H	Europe	RMA	Mean annual precipitation	combined	δ^2^H_f_ = (1.37 × δ^2^H_p_) − 5.52	217	0.67	<0.001	[15]
*Plecotus auritus*	Brown long-eared bat	sedentary	δ^2^H	Europe	LMM / REML	Mean annual precipitation	combined	δ^2^H_f_ = (1.07 × δ^2^H_map_) − 16.84	178	0.72	NR*	[40]
*Plecotus austriacus*	Grey long-eared bat	sedentary	δ^2^H	Europe	RMA	Mean annual precipitation	combined	δ^2^H_f_ = (1.37 × δ^2^H_p_) − 5.52	217	0.67	<0.001	[15]
*Plecotus austriacus*	Grey long-eared bat	sedentary	δ^2^H	Europe	LMM / REML	Mean annual precipitation	combined	δ^2^H_f_ = (1.07 × δ^2^H_map_) − 16.84	178	0.72	NR*	[40]
*Rhinolophus cf. clivosus*	Geoffroy’s horseshoe bat	sedentary	δ^15^N	Africa	OLS	N/A*	combined	^15^N_f_ = (−0.01 × elevation) + 28.78	55	0.32	<0.001	[61]
*Rhinolophus* sp.		sedentary	δ^15^N	Africa	OLS	N/A*	combined	^15^N_f_ = (−0.01 × elevation) + 28.78	55	0.32	<0.001	[61]
*Rousettus aegyptiacus*	Egyptian fruit bat	sedentary	δ^2^H	Africa	RMA	Mean annual precipitation	combined	δ^2^H_f_ = (1.52 × δ^2^H_p_) − 54.09	193	NR*	<0.001	[30]
*Rousettus aegyptiacus*	Egyptian fruit bat	sedentary	δ^13^C, δ^15^N	Africa	OLS	N/A*	combined	elevation = 4635 − (67 × ^15^N_f_) + (112 × ^13^C_f_)	66, 65	0.22	0.004, 0.002	[61]
*Rousettus lanosus*	Long-haired rousette	sedentary	δ^13^C, δ^15^N	Africa	OLS	N/A*	combined	elevation = 4635-(67 × ^15^N_f_) + (112 × ^13^C_f_)	66, 65	0.22	0.004, 0.002	[61]
*Scotophilus dingani*	African yellow bat	sedentary	δ^15^N	Africa	OLS	N/A*	combined	^15^N_f_ = (−0.01 × elevation) + 28.78	55	0.32	<0.001	[61]

“NR*” signifies information that was not reported in the literature source while “N/A*” signifies the information in that column is not applicable. Regression methods are abbreviated in the table as follows: Reduced Major Axis (RMA), Linear Mixed Effects Model fit by Reduced Maximum Likelihood (LMM/REML), Ordinary Least Squares (OLS), Generalized Linear Model (GLM), Linear Mixed Effects Model (LMM).

## Appendix B. Mass Requirements and Analysis Mechanisms for δ^18^O, δ^34^S, ^87^Sr/^86^Sr, and Trace Element/Contaminant Analysis of Modern Tissue Samples

**Table A1:** Mass requirements and analysis mechanisms for δ^18^O analysis of modern tissue samples. Notice the small sample size required for analysis when compared with the other lesser-used intrinsic marking techniques. Additionally, notice the differences in sample size required for calciferous tissues when compared with the others. “NR” signifies information that was not reported in the literature source.

Study Species	Common Name	Tissue Sample	Mass of Sample (mg)	Analysis Mechanism	Reference
*Passer domesticus* L.	House sparrow	Blood (plasma)	0.1–0.2	TC-EA-IRMS	[80]
*Cortunix japonica*	Japanese quail	Blood (plasma)	0.14 ± 0.03	CF-IRMS	[81]
*Passer domesticus* L.	House sparrow	Blood (RBC)	0.1–0.2	TC-EA-IRMS	[80]
*Cortunix japonica*	Japanese quail	Blood (RBC)	0.14 ± 0.03	CF-IRMS	[81]
*Cortunix japonica*	Japanese quail	Body water	0.14 ± 0.03	CF-IRMS	[81]
*Falco sparverius*	American Kestrel	Feather	NR	CF-IRMS	[79]
*Passer domesticus* L.	House sparrow	Feather	0.1–0.2	TC-EA-IRMS	[80]
*Cortunix japonica*	Japanese quail	Feather	0.14 ± 0.03	CF-IRMS	[81]
several species of insectivorous passerines	Passerines	Feather	0.350 ± 0.02	HTC-CF-IRMS	[82]
*Microtus californicus*	California vole	Fur	0.30–0.35	EA-CF-IRMS	[78]
*Cortunix japonica*	Japanese quail	Intestine	0.14 ± 0.03	CF-IRMS	[81]
*Cortunix japonica*	Japanese quail	Liver	0.14 ± 0.03	CF-IRMS	[81]
*Cortunix japonica*	Japanese quail	Muscle	0.14 ± 0.03	CF-IRMS	[81]
*Rangifer tarandus granti*	Alaskan caribou	Tooth enamel	5.0	CF-IRMS	[47]
*Bison bison bison*	Bison	Tooth enamel	3.0–4.0	CF-IRMS	[76]
*Rangifer tarandus*	Caribou	Tooth enamel	1.0–5.0	CF-IRMS	[48]
*Equus cedralensis, E. conversidens, E. mexicanus*	Fossil horses	Tooth enamel	NR	GC/IRMS	[77]

**Table A2:** Mass requirements and analysis mechanisms for δ^34^S analysis of modern tissue samples. Notice the variation in analysis mechanism among studies. Studies denoted with an asterisk (*****) included V_2_O_5_ in addition to the keratin sample in the tin capsule before analysis to aid in sulfate decomposition. The amount of V_2_O_5_ added to the sample varied from 0.1–4 mg depending on the study, although this was not always reported. “NR” signifies information that was not reported in the literature source.

Study Species	Common Name	Tissue Sample	Mass of Sample (mg)	Analysis Mechanism	Reference
*Sus scrofa domesticus*	Domestic pig	Bone collagen	11.0	EA-VisION IRMS	[100]
*Sus scrofa domesticus*	Domestic pig	Faeces	2.0	EA-VisION IRMS	[100]
Several species of raptors	Raptors	Feather	2.0–3.0	EA-CF-IRMS *****	[105]
*Anas platyrhynchos, A. acuta*	Mallard, northern pintail	Feather	1.0–1.8	EA-CF-IRMS	[106]
*Anser albifrons*	Greater white-fronted goose	Feather	NR	3 Element EA-CF-IRMS	[228]
*Anser fabalis fabalis*	Taiga bean goose	Feather	3.5 ± 0.1	EA-IRMS	[111]
Several species of waterfowl	Waterfowl	Feather	3.5	EA-CF-IRMS	[107]
*Eptesicus fuscus*	Big brown bat	Fur	2.0	EA-CF-IRMS *****	[68]
*Nyctalus noctula*	Common noctule	Fur	1.0–1.2	EA-CF-IRMS	[108]
*Microtus californicus*	California vole	Fur	0.9–1.1	EA-CF-IRMS *****	[78]
*Sus scrofa domesticus*	Domestic pig	Hair	2.0	EA-VisION IRMS	[100]
*Bos taurus*	Domestic cattle	Hair	1.0–1.3	EA-VisION IRMS	[110]
*Sus scrofa domesticus*	Domestic pig	Liver	2.0	EA-VisION IRMS	[100]
*Sus scrofa domesticus*	Domestic pig	Milk	2.0	EA-VisION IRMS	[100]
*Sus scrofa domesticus*	Domestic pig	Muscle	2.0	EA-VisION IRMS	[100]

**Table A3:** Mass requirements and analysis mechanisms for ^87^Sr/^86^Sr analysis of modern tissue samples depending on the use of a thermal ionization mass spectrometry (TIMS) or multi collector inductively coupled plasma mass spectrometry (MC-ICP-MS). Notice the variation in mass requirements when using TIMS when compared with the more traditional MC-ICP-MS.

Study Species	Common Name	Tissue Sample	Mass of Sample (mg)	Analysis Mechanism	Reference
*Dendroica caerulescens*	Black-throated blue warbler	Bone	2.0–25	TIMS	[119]
Several species of shorebirds	Bhorebirds	Bone	50–100	MC-ICP-MS, TIMS	[126]
*Taurotragus* spp.	Eland	Bone	14–28	MC-ICP-MS	[132]
Several species of shorebirds	Shorebirds	Feather	50–100	MC-ICP-MS, TIMS	[126]
*Acrocephalus schoenobaenus*	Sedge warbler	Feather	1–2.2	TIMS	[123]
*Tachycineta bicolor*	Tree swallow	Feather	4.8–10.3	MC-ICP-MS	[120]
*Homo sapiens sapiens*	Humans	Fingernail	20–80	MC-ICP-MS	[229]
*Pipistrellus nathusii*	Nathusius’ Pipistrelle	Fur	0.5–5	TIMS	[39]
*Homo sapiens sapiens*	Human	Hair	3.0–7.9	TIMS	[230]
*Homo sapiens sapiens*	Human	Hair	50	MC-ICP-MS	[231]
Rodentia	Several species of rodent	Tooth (whole)	14–28	MC-ICP-MS	[132]
*Rangifer tarandus granti*	Alaskan caribou	Tooth enamel	5.0	PIMMS	[47]
*Bison bison bison*	Bison	Tooth enamel	20	MC-ICP-MS	[76]

**Table A4:** Mass requirements and analysis mechanisms for trace element or contaminant analysis of modern tissue samples. “NR” signifies information that was not reported in the literature source. Notice the widespread lack of reporting; whole mass of sample was often listed, but the final dry mass used for analysis was rarely reported.

Study Species	Common Name	Tissue Sample	Mass of Sample (mg)	Analysis Mechanism	Reference
*Mops condylurus, Tadarida aegyptiaca*	Angolan free-tailed, Egyptian free-tailed bat	Blood	NR	ICP-MS	[155]
*Miniopterus schreibersii*	Common bentwing bat	Bone	NR	ICP-MS	[152]
*Tadarida teniotis*	Free-tailed bat	Bone	NR	ICP-MS	[152]
*Hypsugo savii*, *Nyctalus leisleri*, *Pipistrellus pipistrellus*, *Pipistrellus pygmaeus*	Savi’s pipistrelle, lesser noctule, common pipistrelle, soprano pipistrelle	Bone	NR	ICP-MS	[153]
*Hypsugo savii*, *Nyctalus leisleri*, *Pipistrellus pipistrellus*, *Pipistrellus pygmaeus*	Savi’s pipistrelle, lesser noctule, common pipistrelle, soprano pipistrelle	Brain	NR	ICP-MS	[153]
*Phalacrocorax auritus*	Double-crested cormorant	Feather	NR	CVAFS	[142]
several species of Arctic seabird	Arctic seabirds	Feather	0.5–2.0	Advanced Mercury Analyzer	[144]
*Myotis lucifugus*, *M. leibii*, *M. septentrionalis*, *Eptesicus fuscus*	Little brown, eastern small-footed, nothern long-eared, big brown bat	Fur	NR	ICP-MS, Fixed Wave Mercury Monitor	[149]
Eptesicus fuscus, *Lasionycteris noctivagans*, *Lasiurus cinereus*, *Myotis lucifugus*	Big brown, little brown, silver-haired, hoary bat	Fur	1.0–2.0	Direct Mercury Analyzer	[141]
*Myotis myotis*	Greater mouse-eared bat	Fur	NR	ICP-MS	[151]
*Myotis bechsteinii*, *Myotis nattereri*, *Plecotus auritus*	Bechstein’s, Natterer’s, brown long-eared bat	Fur	NR	ICP-OES	[150]
*Hypsugo savii*, *Nyctalus leisleri*, *Pipistrellus pipistrellus*, *Pipistrellus pygmaeus*	Savi’s pipistrelle, lesser noctule, common pipistrelle, soprano pipistrelle	Fur	NR	ICP-MS	[153]
*Lasiurus borealis*	Eastern red bat	Fur	NR	High resolution ICP-MS	[38]
*Mops condylurus*, *Tadarida aegyptiaca*	Angolan free-tailed, Egyptian free-tailed bat	Fur	NR	ICP-MS	[155]
*Hypsugo savii*, *Nyctalus leisleri*, *Pipistrellus pipistrellus*, *Pipistrellus pygmaeus*	Savi’s pipistrelle, lesser noctule, common pipistrelle, soprano pipistrelle	Heart	NR	ICP-MS	[153]
*Tadarida teniotis*	Free-tailed bat	Kidney	NR	ICP-MS	[152]
*Myotis myotis*	Greater mouse-eared bat	Liver	NR	ICP-MS	[151]
*Tadarida teniotis*	Free-tailed bat	Liver	NR	ICP-MS	[152]
*Hypsugo savii*, *Nyctalus leisleri*, *Pipistrellus pipistrellus*, *Pipistrellus pygmaeus*	Savi’s pipistrelle, lesser noctule, common pipistrelle, soprano pipistrelle	Liver	NR	ICP-MS	[153]
*Tadarida teniotis*	Free-tailed bat	Skin-fur	NR	ICP-MS	[152]
*Tadarida teniotis*	Free-tailed bat	Skinned body	NR	ICP-MS	[152]
*Miniopterus schreibersii*	Common bentwing bat	Whole body	NR	ICP-MS	[152]
*Tadarida teniotis*	Free-tailed bat	Whole body	NR	ICP-MS	[152]
*Hypsugo savii*, *Nyctalus leisleri*, *Pipistrellus pipistrellus*, *Pipistrellus pygmaeus*	Savi’s pipistrelle, lesser noctule, common pipistrelle, soprano pipistrelle	Wing membrane	NR	ICP-MS	[153]
